# On Senders’s Models of Visual Sampling Behavior

**DOI:** 10.1177/0018720820959956

**Published:** 2020-10-07

**Authors:** Y. B. Eisma, P. A. Hancock, J. C. F. de Winter

**Affiliations:** 1 2860 Delft University of Technology, Netherlands; 2 6243 University of Central Florida, USA

**Keywords:** visual attention, computer simulation, sampling, replication, bandwidth

## Abstract

**Objective:**

We review the sampling models described in John Senders’s doctoral thesis on “visual sampling processes” via a ready and accessible exposition.

**Background:**

John Senders left a significant imprint on human factors/ergonomics (HF/E). Here, we focus on one preeminent aspect of his career, namely visual attention.

**Methods:**

We present, clarify, and expand the models in his thesis through computer simulation and associated visual illustrations.

**Results:**

One of the key findings of Senders’s work on visual sampling concerns the linear relationship between signal bandwidth and visual sampling rate. The models that are used to describe this relationship are the periodic sampling model (PSM), the random constrained sampling model (RCM), and the conditional sampling model (CSM). A recent replication study that used results from modern eye-tracking equipment showed that Senders’s original findings are manifestly replicable.

**Conclusions:**

Senders’s insights and findings withstand the test of time and his models continue to be both relevant and useful to the present and promise continued impact in the future.

**Application:**

The present paper is directed to stimulate a broad spectrum of researchers and practitioners in HF/E and beyond to use these important and insightful models.

## Introduction

John W. Senders (1920–2019) left a significant imprint on a number of different areas of human factors/ergonomics (HF/E), including modeling of human error, mental workload, and manual control. Herein, we focus on what is arguably the most impactful element of his scientific career: the topic of visual attention. We review and expand upon the ideas expressed in Senders (1983) doctoral thesis entitled “visual sampling processes.” Senders obtained his formal doctoral qualification later in life with an advisor who had once been his advisee (see Hancock et al., in press, this volume). This thesis reported a culmination of a number of his previous publications including “The human operator as a monitor and controller of multi-degree of freedom systems” (Senders, 1964), “A re-analysis of the pilot eye-movement data” (Senders, 1966), and “The attentional demand of automobile driving” (Senders et al., 1967).

In his thesis, Senders described experiments in which he asked participants to view a bank of dials with randomly moving pointers. This experimental configuration was inspired by the paper “Eye movements of aircraft pilots during instrument-landing approaches” by Fitts et al. (1950). In the latter work, Fitts and his colleagues had concluded that the frequency of eye glances to a particular instrument indicated the relative importance of that instrument. One particular limitation of the Fitts et al.’s study, however, is that it is entirely descriptive, without using a quantitative model. Senders (2016) noted that “psychologists generally shy away from seeing integral signs and partial derivatives in a paper; they just don’t read it” (p. 50:48). In his thesis, he further explained that “The Pilot Eye Movement Studies were being carried out but in a quite nonanalytic way. That lack of analyticity was displeasing” (Senders, 1983, p. 100). Senders, while reflecting on three distinct disciplines in human–machine systems research: human factors, engineering psychology, and the engineering approach, noted: “I’ve been involved in all three” (Senders, 2016, p. 50:43; Hancock et al., 2019). In his thesis, Senders sought to link the different disciplines by introducing a mathematical approach and notation concerning the psychological problem of attention distribution. Through his experiments, he demonstrated a common principle of attentional demand—namely that visual sampling rate toward any one source is linearly related to the bandwidth expressed by that specific source.

In the 1960s and 1970s, Senders’s work sparked a number of follow-up modeling efforts that attempted to extend and refine his work (Carbonell et al., 1968; Carbonell, 1966; Kvålseth, 1978; Sheridan, 1970). As of today, Senders’s observation, and the principle of attentional demand in particular, is more broadly recognized as one of the landmark results in HF/E. Sheridan (2017), in his review of the most impactful HF/E models, identifies Senders’s principle of visual sampling as being amongst them. Similarly, in a more recent review on human performance modeling, Li et al. (2020) categorized Senders’s findings alongside other groundbreaking models such as the Hick–Hyman Law of reaction time and Fitts’s Law concerning the speed and accuracy of human movement. Wickens (2008) subsequently developed a now well-known model of visual information sampling called the salience, effort, expectancy, value (SEEV) model. For the development of the “expectancy” component of this model, Wickens relied extensively on Senders’s pioneering work. More specifically, Wickens defined the element of expectancy directly in terms of bandwidth or event rate.

With respect to the above observations, it is reasonable to conclude that Senders’s work is highly regarded in HF/E as well as in scientific areas beyond. However, as with many classics, it may be that it is more often cited than actually read. With certain exceptions (e.g., Moray, 1986), Senders’s work on visual sampling is frequently cited in more of a “generic” manner without referring to either its assumptions or its mathematical intricacies. Senders’s work presents various equations, but hardly any visual illustrations or graphic examples that would make his work more immediately accessible to a broader audience. Another concern is that Senders performed his experiments using only a small number of participants (typically about five). In the wake of the replication crisis in psychology (Loken & Gelman, 2017), this small number has now become some cause for concern. Accordingly, there now emerges a contemporary need for an accessible yet critical review of Senders’s models, his assumptions, and his results. Herein, we look to explain and clarify Senders (1983) exposition of his models through relevant computer simulations and elaborative visual illustrations. We strive to facilitate an understanding of these models so that a wider spectrum of researchers in HF/E and beyond will be able to use them in their own research endeavors.

## Task and Pointer Signals

**Figure 1:**
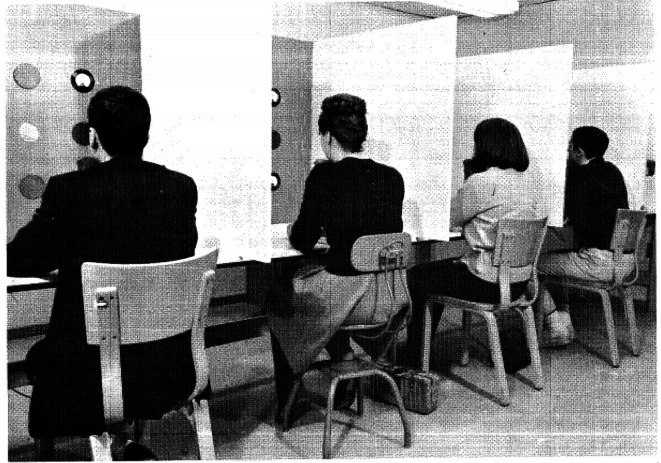
Experimental setup used by Senders (Senders et al., 1966).

As noted, Senders asked his participants to watch a bank of dials, each dial containing a pointer that moved in a predefined manner. Figure 1 illustrates this experimental setup, consisting of a number of separate booths. Participants were to press a button when any of the pointers exceeded a predetermined threshold value on either side of the dial. Although Senders used a small number of participants, he did record extensive data from them across numerous sessions; thus, “three min of camera time were obtained at the beginning and at the end of each one h session. There was one session on each of 30 successive days” (Senders, 1983, p. 42).

The signals that drove the pointers, which were different for each dial, were defined using a technique that makes pointer deviations subjectively unpredictable, while its actual overall movement characteristics are known. This technique, which has also been used in manual control research, involves the summation of sine waves of different frequencies with random phase shifts (e.g., McRuer & Jex, 1967). The summation technique used to obtain the pointer signal of dial *i* as a function of time, $y_{i}(t)$ , is described in Equation (1), in which *k* is the current sinusoid number, *m* is the number of sinusoids, $\Omega_{k}$ is the frequency of the sinusoid in Hz, *t* is time, and $\theta_{k}$ is a random phase shift for sinusoid *k*.

(1) $$y_{i}\left(t\right)=\sum_{k=1}^{m}sin\left(2\pi {\text{Ω}}_{k}t+\theta_{k}\right)$$

**Figure 2:**
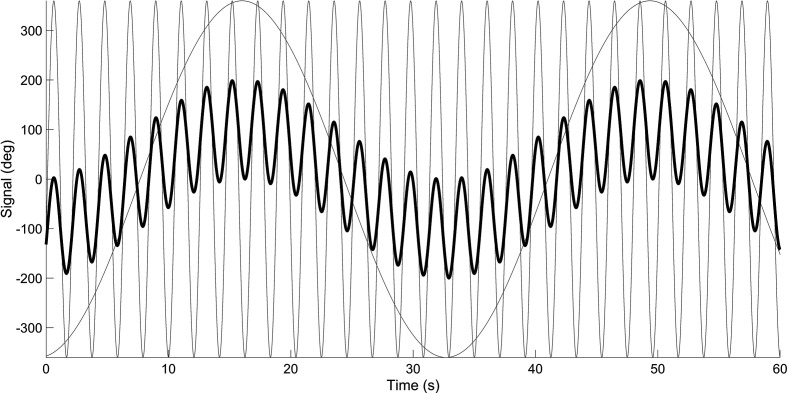
Two sinusoids (in gray) and the summed signal (in black). The summed signal has been divided by a constant so that the standard deviation equals 100°.

**Figure 3:**
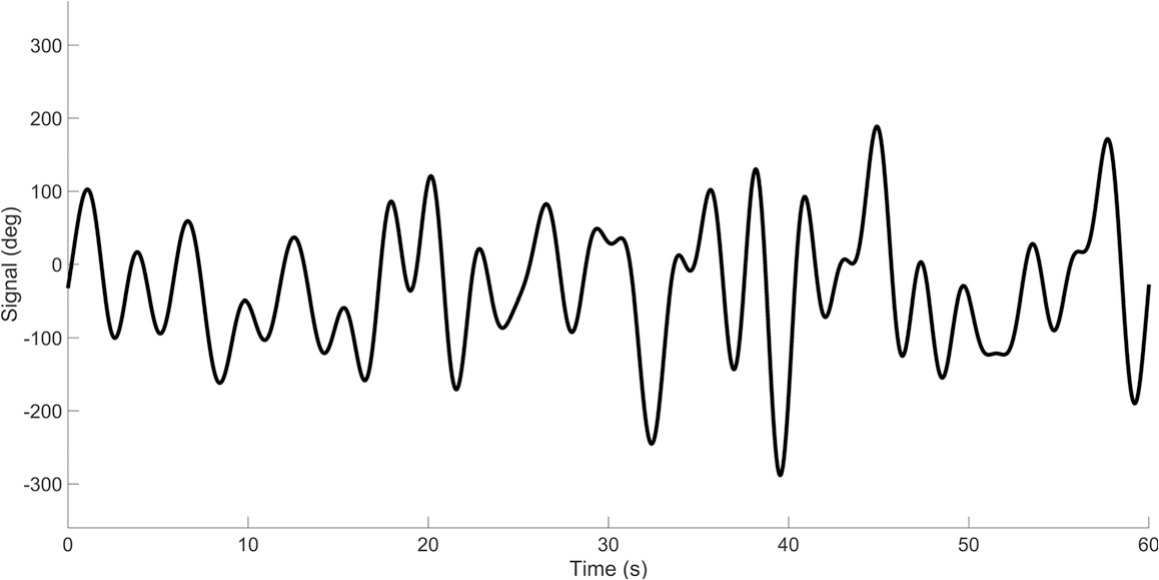
Signal having a bandwidth of 0.48 Hz. The signal is the sum of 1000 sinusoids. The summed signal has been divided by a constant so that the standard deviation equals 100°.

Figure 2 shows a representation of 2 signals (*m* = 2), having frequencies of 0.03 Hz and 0.48 Hz, respectively. It can be seen that the sum of the 2 signals has a predictable waveform. However, by sequentially increasing the number of signal components *m*, the signal becomes progressively more unpredictable to the human observer. The signal in Figure 3 represents the summation of 1000 sine waves, with frequencies between 0.001 and 0.48. The summed signal of dial *i* is said to have a “bandwidth” or cutoff frequency of 0.48 Hz, *W_i_* = 0.48 Hz, meaning that the signal is composed of a 0.48-Hz-wide band of frequency components.

In this example, the frequencies were spaced linearly between 0.001 and 0.48 but another option is to use logarithmic spacing (Damveld et al., 2010; Eisma et al., 2018). We opted for 1000 sinusoids to illustrate that the concept of multi-sine creation works even when the number of sinusoids is extremely high, and to ensure that all frequencies are appropriately represented. In comparison, Senders (1983) reported that “each meter was driven by a signal, as used by Elkind (1956), composed of more than 40 sinusoids” (p. 57). Elkind (1956), in turn, reported summing between 40 and 144 sinusoids, and claimed: “Although 40 (the smallest number of components used frequently in these experiments) is not a very large number, it is large enough so that no periodicities in the signals are obvious” (p. 12).

In his experiments, Senders presented participants with either four or six identical dials, each having a different signal bandwidth. A low-bandwidth signal appears as a slowly moving pointer and should hypothetically require little relative attention for detecting critical events, that is, detecting whether the pointer angle exceeds the threshold value. A high-bandwidth pointer, it was hypothesized, demands more attention.

## Nyquist–Shannon Sampling Theorem

**Figure 4:**
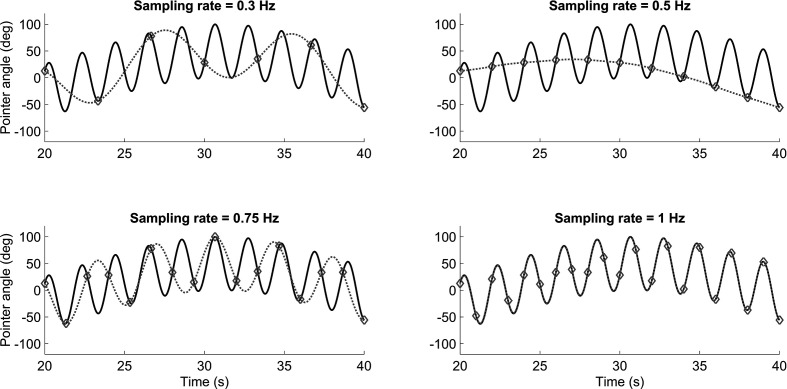
Illustration of the Nyquist–Shannon sampling theorem. If sampling at a frequency that is more than twice the bandwidth, signal reconstruction is possible. The gray dotted line represents the signal that is reconstructed from the samples. The samples are represented by the markers.

Senders was concerned with predicting how often an “ideal human observer” ought to visually sample each of the dials. He explains his Eureka moment that led him to a solution: “upon re-reading Shannon (3) … I experienced a sudden awareness of the significance of the sampling notion for the understanding of the visual scanning behavior of human beings” (Senders, 1983, p. 100). More specifically, Senders conceived of adapting the Nyquist–Shannon sampling theorem for predicting human visual sampling behavior. The Nyquist–Shannon sampling theorem states that if one were to seek to sample a signal without losing information, one must sample (i.e., take an observation) at a frequency that is at least twice the bandwidth (i.e., the highest frequency in the signal). This theorem is illustrated in Figure 4, which shows, in black, a signal consisting of two sinusoids having frequencies of 0.03 Hz and 0.48 Hz, respectively. If one samples at 0.3 Hz, that is, if taking a reading only 0.3 times per second, it is effectively impossible to reconstruct, and thus accurately respond to the signal. The situation does not improve when sampling at 0.5 Hz or even 0.75 Hz, but when sampling at 1 Hz, which is more than twice the highest frequency (0.48 Hz) of the signal, then perfect signal reconstruction becomes possible. In these simulations, the signal was reconstructed using the Whittaker–Shannon interpolation formula (Matthé, 2017).

## Periodic Sampling Model

**Figure 5:**
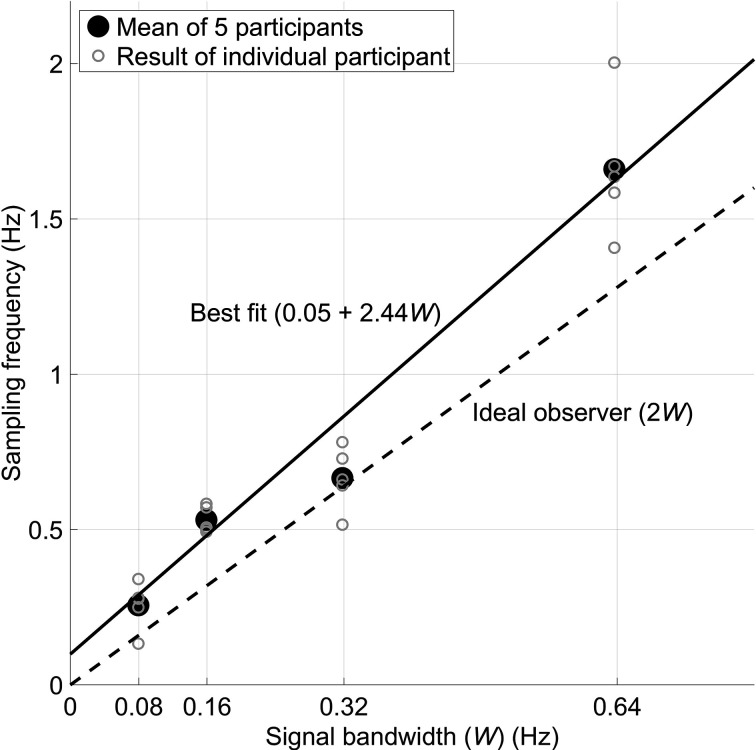
Senders’s results show a convincingly strong correlation (*r* = 0.98 at individual and aggregate levels) between bandwidth and visual sampling. Five participants watched four dials and pressed a button when any of the four signals exceeded a threshold angle. Eye movements were recorded using a motion picture camera.

Thus, Senders hypothesized that human operators behave as ideal observers who attempt to reconstruct the observed signals. To do this, the human would have to periodically sample the signal, just as shown in Figure 4. We should caution that Senders did not believe that humans actually act as periodic samplers: “The periodic sampler was originally constructed as a simple and *unrealistic* model of human behavior” (Senders, 1983, p. 37; emphasis ours). That is, it is rather unlikely that humans act to formulate perfect knowledge of the signal characteristics and then sample at a fixed frequency that is entirely independent of the momentary pointer angle and its velocity. However, Senders was sanguine about his use of the Nyquist–Shannon Theorem as a foundation for his models. He then produced evidence that human operators do behave in strong accordance with such a sampling criterion. Figure 5 (redrawn from Senders’s thesis, p. 51) shows that participants actually sampled at a frequency (2.44*W*, *W* being the bandwidth of the pointer signal), just above the Nyquist rate (*2W*).

In his periodic sampling model (PSM), Senders defined the amount of attention that each dial required. More specifically, the attentional demand ( $T_{i}$ ) of a particular dial was defined as two times the product of the signal bandwidth ( $W_{i}$ ) and the sampling duration ( $D_{i}$ ) for that dial:

(2) $$T_{i}=2W_{i}\times D_{i}$$

For example, if the signal bandwidth was 0.48 Hz, and the sampling duration is held constant at 0.30 s, which would be a typical fixation duration (e.g., Rayner, 1998), then $T_{i}$ is 0.288. This would mean that, if a sampling task lasts, for example, 100 s, dial *i* would be expected to absorb 28.8 s of attention.

The attentional demand for all dials combined is

(3) $$T_{sum\;=\;\sum_{i=1}^{m}T_{i}}$$

where *m* specifies the number of dials.

Using Equation (3), it is eminently possible to then guide the design of the human–machine interfaces. For example, if there are currently *m* dials (or displays/tasks) and there arises the need to add another (*m* + 1)-th, this becomes possible only if $T_{sum}\;+\;T_{m+1}\;\leq \;1$ . Otherwise, adding that demand overwhelms the human observer.

The attentional demand as computed in Equations (2) and (3) represent ideal values. In reality, humans prove to be rather less efficient, and the combination of signal bandwidths may inhibit periodic sampling. As explained by Senders, it is unlikely that periodic sampling is feasible when there is more than one dial:

Only in extremely rare circumstances would it be possible for strictly periodic sampling to take place on a multitude of instruments in an operational task. The periods would have to be commensurable and of such size as to permit a repeated sequence to occur. (p. 28)

## Random Constrained Sampling Model

In response to the above observation, Senders proposed an alternative model, which he called the random constrained sampling model (RCM). This model proves similar to the PSM since it assumes that the human operator samples the dials according to their bandwidths. However, the RCM assumes that the human is otherwise ignorant and samples the instruments entirely randomly instead of periodically. In the RCM, the probability that instrument *i* is sampled ( $p_{i}$ ) equals the bandwidth of the signal relative to the total bandwidth of all signals. For example, if the human operator is looking at four dials having bandwidths of 0.08, 0.16, 0.32, and 0.64 Hz, respectively, then the sampling probabilities, $p_{i}$ , of these four dials would be 6.7, 13.3, 26.7, and 53.3%, respectively); see Equation (4).

(4) $$p_{i}=\frac{W_{i}}{\sum_{i=1}^{m}W_{i}}$$

Predicting the sampling interval for a particular dial *i*, under the assumption that each dial is sampled independently, can be accomplished using the geometric distribution shown in Equation (5). Here, $p_{i}$ is the probability that a sample of the human operator falls on a dial *i*—as defined in Equation (4)—and $P_{r}\left(X_{i}=k\right)$ denotes the distribution of the probability that the *k*-th sample (*k* = 1, 2, 3, …) of the human operator falls on dial *i* for the first time.

(5) $$P_{r}\left(X_{i}=k\right)={\left(1-p_{i}\right)}^{k-1}p_{i}$$

For example, suppose that $p_{i}$ = 13.3%, then the probability is 13.3% that the human operator first samples dial *i* on the first occasion (*k* = 1), the probability is 11.6% that the human operator first samples dial *i* on the second occasion (*k* = 2), the probability is 10.0% that the human operator first samples dial *i* on the third occasion (*k* = 3), and so on. This distribution converges to 0 for large *k*, because it becomes increasingly likely that dial *i* has already been sampled.

If we assume a fixed sampling duration D for all dials (an assumption that can be justified according to Senders, because the required precision of reading is the same for all dials; Senders, 1983, p. 29), then the expected value of the time interval between two successive fixations on any particular dial becomes (e.g., Dekking et al., 2005, p. 153):

(6) $$\begin{array}{rl} \mu_{interval,i} & =1Dp_{i}{\left(1-p_{i}\right)}^{0}\\ & +2Dp_{i}{\left(1-p_{i}\right)}^{1}+3Dp_{i}{\left(1-p_{i}\right)}^{2}+\ldots =\frac{D}{p_{i}} \end{array}$$

**Figure 6:**
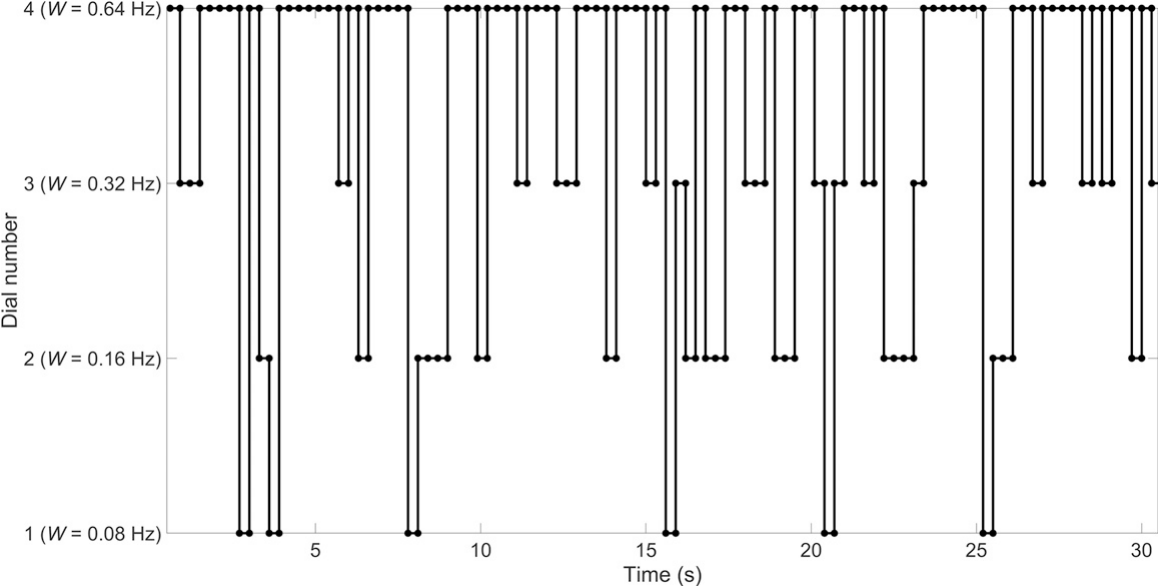
Example of 30 s of random sampling for a fixation duration of 0.3 s, and sampling probabilities of 6.7%, 13.3%, 26.7%, and 53.3% for Dials 1, 2, 3, and 4, respectively.

For example, if a dial is sampled with a probability of 13.3% ( $p_{i}$ = 13.3%) and the sampling duration D is 0.3 s, then the expected sampling interval for dial *i* is 2.25 s. An illustration of these sampling efforts is provided in Figure 6, shown here for a four-dial instrument panel. In this illustration, Dial 2 is sampled on average every 2.25 s.

Senders (1983) also made predictions about the sampling duration of each instrument. Up until this point, we have assumed a fixed sampling duration, *D*. However, as shown in Figure 6, due to the occasional repeated sampling of the same dial, the observed *D* proves to be higher than 0.3 s. The correction factor used by Senders is specified in Equation (7).

(7) $$\bar{D_{o}}=D\frac{1}{1-p_{i}}$$

The outcome of this correction results for Dial 2, for example, in the average observed fixation duration, $D_{0}$ , to be 0.346 s rather than 0.3 s.

## Conditional Sampling Model

The RCM assumes that the human samples the dials based on bandwidth only. This would imply that the human, after practice, has formed some type of mental model of the bandwidths of the dials. Senders (1983, p. 85-86) stated that:

The decision for the practiced observer is hardly in the nature of a voluntary one. … Rather it is as if the eyes’ mind, earlier hypothesized, directed the eyes in such a way as to bring to attention what the mind’s eye wanted to see.

In practice, however, human operators may sample a dial not only based on bandwidth but also based on the absolute position of the pointer and/or its velocity. Senders (1983, p. 3232), in typical fashion, illustrated this through the use of a metaphor of a baby crawling in the vicinity of a swimming pool:

Imagine yourself to be trying to read this monograph whilst seated on the lawn near a swimming pool. An infant is crawling on the grass generating a ‘random crawl’. You wish to intervene when the infant is likely to fall into the pool and you wish to get as much reading done as possible, as well. A sensible strategy would be to calculate a next time to look at the infant based on what you had observed on the last look. If the infant had been close to the pool’s edge, you would look much sooner than if it had been far away. Other things being equal, you would look sooner if it had been approaching the edge of the pool than if it had been receding from it. Lastly, in general and other things being equal, you would look sooner if it were an active infant than if it were a lethargic one. Thus the determinants of your observing behavior would be: the amount by which the value observed fell short of the limit, the derivative of the observed variable, and the mean absolute velocity of the variable (which will be a direct function of the bandwidth of the signal formed by the positions of the infant in time).

**Figure 7:**
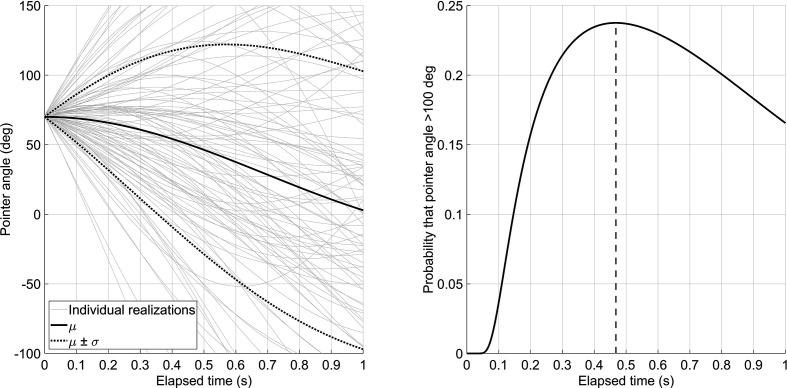
Left: Expected value, $\mu_{{\hat{y}}_{i}\left(t\;+\;\tau \right)|y_{i}\left(t\right)\;=\;Y_{i}},$ and corresponding standard deviation of the predicted future signal, $\sigma_{{\hat{y}}_{i}\left(t\;+\;\tau \right)|y_{i}\left(t\right)\;=\;Y_{i}},$ for elapsed time (τ) since taking a reading $Y_{i}$ of 70°. Also shown are a random 100 realizations of the pointer angle with $Y_{i}$ ≈ 70°. Right: The corresponding probability that the pointer angle exceeds 100°; the probability has a maximum at *T* + τ = 0.47 s. The pointer signal has a 0.48-Hz bandwidth and overall standard deviation of 100° (see Figure 3 for an example of the signal).

As a consequence, in his thesis, Senders thus proposed a conditional sampling model (CSM). The essence of this is that the human operator samples the dials based on uncertainty. A key variable in this model is the autocorrelation of the signal displayed by the pointer, that is, the correlation of the signal with a time-delayed copy of itself. The autocorrelation ρ equals 1 if the time shift τ is 0 s. The autocorrelation theoretically drops with increasing τ. The idea of this time-dependent uncertainty is illustrated in Figure 7. Suppose that, at a given moment, *t*, the human observes dial *i* having a pointer angle of 70° ( $y_{i}(t)=Y_{i}=70^{\circ }$ ). If the human resamples dial *i* shortly afterwards, for example, at *t* + τ = 0.1 s, then the pointer is most probably still close to that 70° value. The expected value of the pointer angle with respect to time, given an initial reading $Y_{i}$ , is provided by Equation (8), where $\rho_{i}\left(\tau \right)$ is the autocorrelation function of the signal (Senders et al., 1966, p. 23). It is noted that Equation (8) assumes that the operator reads only the current pointer angle, $Y_{i}$ , not the pointer velocity. Better predictions will be possible, and the operator will therefore need to sample less often, if he not only reads pointer angle but also pointer velocity (Fogel, 1955).

(8) $$\mu_{{\hat{y}}_{i}\left(t+\tau \right)|y_{i}\left(t\right)=Y_{i}}=\rho_{i}(\tau )Y_{i}$$

For a bandlimited white noise signal with linearly spaced frequencies (Figure 3), the autocorrelation function, $\rho_{i}(\tau )$ , is known to be (Knudtzon, 1949, p. 6; Senders, 1983, p. 33):

(9) $$\rho_{i}\left(\tau \right)=\frac{sin\left(2\pi W_{i}\tau \right)}{2\pi W_{i}\tau }$$

So, suppose the signal bandwidth $W_{i}$ is 0.48 Hz, then the expected value of the pointer angle for τ = 0.1 s will be 68.9° or very close to the original 70°. As τ increases, the initial reading of 70° becomes less and less influential. For example, if τ = 1.0 s, then the expected value is 2.9°, that being much closer to the overall expected value of 0° (see the solid line in Figure 7, left).

The standard deviation of the predicted future signal, ${\hat{y}}_{i}(t+\tau )|y_{i}(t)=Y_{i}$ , can be computed using Equation (10); see Senders (1983, p. 34). This standard deviation is a measure of the uncertainty of the prediction, where it is noted that $1-\rho_{i}^{2}(\tau )$ is the fraction of the variance in the predicted future signal that is uncorrelated with $y_{i}\left(t\right)$ .

(10) $$\sigma_{{\hat{y}}_{i}}(t+\tau )|y_{i}(t)=Y_{i}=\sigma_{y_{i}}\sqrt{\left(1-\rho_{i}^{2}(\tau )\right)}$$

At the moment of sampling the dial, the human is entirely certain about the status of the signal. That is, $\sigma_{{\hat{y}}_{i}\left(t+0\right)}|y_{i}\left(t\right)=Y_{i}$ = 0°. The longer the operator does not sample the dial, the higher the uncertainty. If τ is 0.1 s and the standard deviation of the entire signal $\left(\sigma_{y_{i}}\right)$ is 100°, then $\sigma_{{\hat{y}}_{i}\left(t\;+\;0.1\right)|y_{i}\left(t\right)\;=\;Y_{i}}$ is 17.3°, which is effectively small. The standard deviation rises to its nominal value of 100° as τ increases. This is illustrated in Figure 7 (left), in which the standard deviation of the predicted pointer angle is the distance between the solid mean line and the two dotted lines. The rise of uncertainty about the value of the signal is analogous to the metaphorical crawling infant. If the parent has not seen the infant for a while, then logically, the parent should be ever-more concerned about whether the infant has fallen into the pool.

Let us now assume that the operator is tasked to press a button whenever the pointer exceeds a critical value $L_{i}$ . We can, from the foregoing observations, calculate the probability that this pointer angle exceeds the critical value (one-sided), using the normal cumulative distribution function ( Φ ), as shown in Equation (11) (see Senders, 1966, 1983). For example, assume, $L_{i}$ = 100°, $Y_{i}$ = 70°, and $\sigma_{y_{i}}$ = 100°. Then, at τ = 0.1 s, the probability of the pointer exceeding the 100° critical value is 3.6% (Figure 7, right). In other words, one-tenth of a second after the initial reading, the probability is only a relatively small one that the pointer is actually exceeding the threshold. Given this understanding, the operator may be disinclined to sample the dial again.

(11) $$\begin{array}{rl} P_{{\hat{y}}_{i}(t+\tau )>L_{i}|y_{i}(t)=Y_{i}} & =1-\Phi \left(\frac{L_{i}-\mu_{{\hat{y}}_{i}\left(t+\tau \right)|y_{i}(t)=Y_{i}}}{\sigma_{{\hat{y}}_{i}(t+\tau )|y_{i}(t)=Y_{i}}}\right)\\ & =1-\Phi \left(\frac{L_{i}-\rho_{i}(\tau )Y_{i}}{\sigma_{y_{i}}\sqrt{\left(1-\rho_{i}^{2}(\tau )\right)}}\right) \end{array}$$

Senders (1983) proposes various types and forms of CSMs in his thesis. For example, in CSM-1, the operator is assumed to sample the dial when the probability of exceeding the critical value is maximal (0.47 s in Figure 7). In CSM-2, the operator samples the dial when the probability of exceeding the critical value is greater than any particular specified probability threshold. The essence of CSM models is that the sampling frequency does not depend on bandwidth alone ( $W_{i}$ , that is, whether the infant is lethargic or not) but also on the last pointer reading ( $Y_{i}$ ; thus, where the infant was in relation to the swimming pool when last seen).

## Modern Replication of Senders’s Experimental Findings

As we noted previously, Senders (1983) used only small numbers of participants (Figure 5). In line with the recent popularity of replication research (Zwaan et al., 2018), Eisma et al. (2018) performed just such a replication and expansion of Senders’s six-dial study, using Senders’s own specified bandwidths of 0.03, 0.05, 0.12, 0.20, 0.32, and 0.48 Hz. The replication study was, however, conducted using a total of 86 participants. The results revealed a remarkably strong and gratifying congruent outcome with Senders’s original results. More specifically:

- Both Senders (1983) and Eisma et al. (2018) found that participants’ mean sampling rate was proportional to bandwidth. More specifically, Eisma et al. (2018) found the following best fit: *Sampling rate* = 0.64*W +* 0.20 (r = 0.98), whereas Senders had earlier found that *Sampling rate* = 0.61*W* + 0.18 (*r* = 0.99). Note that the slope of approximately 0.64W is considerably shallower than the slope of 2.44W , as observed for the four-dial task (Figure 5). This, we believe, is because the six-dial task was attentionally more demanding, the result of which was that participants were unable to distribute their attention optimally.
- Eisma et al. (2018) found an increase of glance duration as a function of dial bandwidth. Senders had originally predicted this effect—see Equation (7)—but the empirical data in Senders’s thesis were indeterminate here, again, perhaps due to the reliance on a small number of participants and the low eye-movement data acquisition rate (12 fps) of his equipment’s measurement capacities at that time. Thus, we have not simply confirmed some of Senders’s original findings but have established the veracity of one of his predictions that, due to inherent constraints, he was not able to fully evaluate this in this own experimentation. More specifically, Eisma et al. found the following best fit: *Mean glance duration* = 0.43*W* + 0.38 (*r* = 0.99), whereas Senders had earlier reported a weaker fit via the specification that *Mean glance duration* = 0.30*W* + 0.47 (*r* = 0.81).
- Senders (1983) was unable to fully test the CSM, but manually annotated participants’ eye movements based on camera images recorded. He had no way of relating these camera recordings directly to the current pointer angle. The replication study of Eisma et al. (2018) used modern eye-tracking equipment with synchronous data recordings in order to be able to accomplish this. The latter authors found, in agreement with the crawling infant analogy, that (1) bandwidth, (2) pointer angle, that is, how close to the threshold it was, and (3) pointer velocity (higher velocities attracting more attention) each strongly influenced the probability that a participant then glanced at a specific dial.

In addition to these encouraging findings, Eisma et al. (2018) noted several points where Senders (1983) may have potentially erred or provided only an incomplete explanation.

- Senders did not specify how the dials were arranged on the instrument panel. For example, if the high-bandwidth dials were placed in the middle, then the associated visual sampling effort might be relatively low. In contrast, if the high-bandwidth dials were positioned toward the edges, then visual sampling effort could have been relatively higher since the human operator would then need to scan across greater distances. Eisma et al. (2018) found that this “effort configuration” does matter, with less ideal sampling (i.e., lower than the Nyquist rate of 2W ) when the needed effort level was higher.
- Senders claimed that people need extensive practice: “So I trained my subjects for more than 30 hr and took data along the way in order to find out how long it took for them to stabilize in their scanning behavior. Indeed, it took about ten hours for scanning to stabilize and more nearly twenty-five for detection to arrive at a reasonable high level” (Senders, 1983, p. 101). Eisma et al. (2018) found, however, that after only 20 s on the task, a clear distinction appeared between the sampling rates for the different dials. This finding suggests that conditional sampling, that is, sampling based primarily or even exclusively on bottom-up sensory cues, represents the dominant psychological mechanism employed by operators.
- Senders (1983) required operators to detect threshold crossings but did not report on any performance data per se. De Winter et al. (2019) showed a strong correlation between participants’ sampling behavior and their detection performance (*r* = 0.78). In other words, people who showed superior sampling (i.e., looking at the right dial at the right time) detected more threshold crossings. Although this might appear to be even self-evident, such a clear link between the spatial orientation of attention and subsequent detection efficiency appears not to have been demonstrated before. For, elsewhere, it has been determined that looking (i.e., the fixation of the eyes) does not necessarily equate to seeing (i.e., the processing of information in that fixated area; and see Krueger et al., 2019).

## Discussion

In this paper, we have reviewed several facets of John Senders’s collective work “Visual Sampling Processes.” Our goal was to make this important work accessible to a broader audience. We have therefore provided illustrations of (1) how to create a random-appearing pointer signal, and what it means to (2) sample periodically in order to reconstruct that pointer signal, (3) sample randomly in a bandwidth-constrained manner, or (4) sample conditionally based on pointer value during the last sample of the dial. Furthermore, we reviewed a recent replication study that demonstrated that the findings of Senders readily replicate. The latter experimental study also validated several of Senders’s predictions that he himself was unable to test with the equipment of his time. Overall, our treatise is intended to recognize Senders’s legacy and to show how his ideas remain relevant to many modern applicational contexts.

We might ask why Senders’s work was so readily replicable? We suggest two major reasons. First, Senders’s empirical findings and untested predictions were based upon substantive models and calculations. As pointed out by Box (1976, p. 792), “all models are wrong”. By this, he meant that all models, in their attempt to make accurate predictions, rest on assumptions and therefore cannot predict the real world *exactly*. All models are necessarily reductions of the world that they seek to portray and so must be, at best, only reduced approximations. In the case of Senders’s models, it is unlikely that humans would sample periodically or randomly without any consideration as to the state of the dials they are viewing. It is also unlikely that humans can flawlessly estimate the probability that the pointer angle would exceed a target threshold angle. Furthermore, according to the aforementioned Wickens’s (2008) SEEV model, there is more to sampling than expectancy and salience alone. “Value” (the cost of not sampling a particular dial) and “effort” (the amount of eye movement and head movement required, as explained previously in the modern replication of Senders) also each affect eye movements. Regardless of these assertions and assumptions, Senders’s models do provide a plausible and useful basis for predicting human sampling behavior. Sheridan (2002) referred to these type of models as “borrowed engineering models.” That is, in and around the 1960s, HF/E researchers started to deviate from purely descriptive “knobs and dials” research, such as the Fitts et al. (1950) studies, and started using the then-available quantitative models. These models were perhaps naturally “borrowed” from the engineering domain since that discipline possessed the most relevant and applicable ones at that time. In Senders’s case, this was from Shannon’s work on information theory (see Shannon & Weaver, 1949). By promulgating these theoretical bases, more realistic predictions of sampling behavior can be made, as compared to purely descriptive approaches. As Senders (2001) put it:

I went back to Shannon, the 1947 article, read the thing again, and decided that the Sampling Theorem would be the controlling factor. Irrespective of what people wanted to do, what they could do, the limitations would be mathematically defined.

A second reason for the high degree of replicability is that Senders did not rely on null-hypothesis significance testing. Probability estimates are nowhere to be found in his work. Instead of performing assessments predicated upon which condition yields significantly different results from comparative and control conditions, Senders estimated functional relationships between experimental variables, for example, between sampling rate and bandwidth. In recent work, Smith and Little (2018) have explained why this type of approach to psychological research is expected to render such replicable results. They showed, through theoretical argument and computer simulation, that high replicability can be achieved even when only a small number of participants are subjected to a large number of trials. This approach corresponds to typical methods in psychophysics (Smith & Little, 2018).

Now that technology is becoming increasingly automated, the human operator is often only a supervisor rather than a direct controller (Hancock, 2014; Sheridan, 2002). For example, in automated car driving, the driver does not necessarily have to turn the steering wheel or press any of the foot pedals. However, presently, drivers still have to monitor the road and occasionally retake vehicle control (Eriksson & Stanton, 2017; Zhang et al., 2019). Since active manual control is absent in automated driving, there is an increasing research focus on indirect control, for example, gestural control and monitoring. Here, the human has to monitor the automation that can, in its turn, monitor the human. Perhaps not surprisingly, in a few years, driver drowsiness/attention monitoring systems will be obligatory in newly sold passenger cars in the European Union (European Commission, 2019). We expect Senders’s work to become increasingly more relevant in such human–automation interaction on both research and application fronts. In his work, Senders used visual occlusion methods to determine the attentional demands of drivers (Saffarian et al., 2015; Senders, 1964). Instead of merely detecting whether drivers are visually attentive or distracted, a computational model could be used to determine whether the driver has sampled the relevant objects in the driving environment (De Winter et al., 2019). We anticipate that Senders’s models will here represent a useful starting point to such computational models. It may be possible, for example, to provide a warning if driver sampling behavior deviates significantly from expectations as determined from signal bandwidth. More specifically, we can postulate that drivers do not have to distribute their attention uniformly across their ambient working environment, but mainly have to look at regions in the visual field where activity is taking place. In other words, human drivers will be obliged to look a lot at the roadway and mirrors, and also at the dashboard, and much less at the scenery or parked cars. As derived from the SEEV model (Wickens, 2008), a separate computational module will have to determine which road regions have high value, and the higher values will have to be assigned to objects that have a higher probability of colliding with the driver’s car.

Our final point on attention concerns the interplay between top-down (bandwidth-based) and bottom-up sampling (pointer angle, velocity) processes. We can affirm and confirm that both factors are relevant, but it remains unknown at present exactly how they jointly contribute to human sampling (and see Hancock, 2019). A high-bandwidth display may attract attention because operators perceive something moving rapidly in their peripheral vision. However, perhaps after an extended period of observation (minutes or even hours), the operator can form expectancies about where to look predicated upon their accumulated situational experience and not just the momentary dynamics of the display(s) before them (e.g., “the left top dial requires most of my attention”). The interplay between the top-down cues (expected value) and bottom-up cues (salient features such as a fast-moving dial) obviously requires further research, an endeavor that, we believe, Senders would be in wholehearted agreement with. Such research may be performed using a gaze-contingent sampling paradigm, in which peripheral vision is occluded. In conclusion, we have looked to give a brief encomium for, and support of, Senders’s models of visual attention. We anticipate that the work of Senders will remain relevant to HF/E research and in realms beyond for many years to come.

## Key Points

- John Senders had a large impact on the science of human factors and ergonomics, especially in the area of visual attention modeling.
- His research findings and models can benefit from explanation, validation, and further dissemination.
- The ideas behind the models used in Senders’s doctoral thesis are reviewed by means of computer simulations and associated graphic illustrations.
- Senders’s results are replicable, and his findings continue to be relevant and impactful.
